# Sildenafil is a candidate drug for Alzheimer’s disease: Mechanism‐of‐action observation in patient iPSC‐derived brain organoids and a 5xFAD mouse model

**DOI:** 10.1002/alz70859_105583

**Published:** 2025-12-26

**Authors:** Yichen Li, Dhruv Gohel, Andrew A. Pieper, Jeffrey L. Cummings, Feixiong Cheng

**Affiliations:** ^1^ Cleveland Clinic, Cleveland, OH USA; ^2^ Brain Health Medicines Center, Harrington Discovery Institute, University Hospitals Cleveland Medical Center, Cleveland, OH USA; ^3^ Chambers‐Grundy Center for Transformative Neuroscience, Department of Brain Health, School of Integrated Health Sciences, University of Nevada Las Vegas, Las Vegas, NV USA; ^4^ Case Western Reserve University, Cleveland, OH USA; ^5^ Cleveland Clinic Genome Center, Cleveland, OH USA

## Abstract

**Background:**

We previously identified Sildenafil (a phosphodiesterase type 5 (PDE5) inhibitor) as a candidate drug in Alzheimer’s Disease (AD) using an in silico network medicine approach. However, the clinically meaningful effect size and mechanism‐of‐action of sildenafil in AD treatment is still unclear.

**Method:**

To further investigate the real‐world effect of sildenafil on AD incidence, we conducted new patient data analyses using both the MarketScan® Medicare with Supplemental database (n=7.23 million older subjects) and the OPTUM database (n=11.52 million older subjects). We utilized AD patient‐induced pluripotent stem cells (iPSC)‐derived neuron and cerebral organoid models (both female and male familial and sporadic AD patients) to explore the mechanism‐of‐action of sildenafil’s protective effects on AD. Additionally, we treated 5xFAD, a transgenic AD mouse model, with 15 mg/kg sildenafil for 3 months. Finally, we conducted behavioral tests, immunofluorescence staining on mouse brain sections, and single‐cell RNA‐seq to evaluate sildenafil’s efficacy and brain target engagement.

**Result:**

Sildenafil usage is significantly associated with reduced likelihood of AD across all four new drug cohorts (bumetanide, furosemide, spironolactone, and nifedipine). Compared to spironolactone, sildenafil was linked with a 46% and 30% reduced prevalence of AD in MarketScan (HR = 54%, 95% CI 0.32‐0.66, *p*‐value = 3.33x10‐5) and OPTUM (HR = 70%, 95% CI 0.49‐1.00, *p*‐value = 0.05), respectively. In AD patient iPSC‐derived neuron models, sildenafil treatment reduces phosphorylated tau (pTau181 and pTau231), phosphorylated GSK‐3b, and CDK5. Sildenafil treatment in mice significantly improved novel object recognition and performance on the Morris water maze and contextual fear conditioning test. Immunostaining revealed significantly decreased amyloid plagues in the isocortex and interbrain of the sildenafil‐treated mice, supporting its therapeutic effects in AD from real‐world patient data and iPSC model findings. RNA‐seq analysis (bulk and single‐cell) identified brain target engagements and potential cell type‐specific pharmacodynamics pathway for potential treatment of AD.

**Conclusion:**

Evidence from real‐world data, patient iPSC‐derived cellular and brain organoids, and in vivo transgenic mouse model observations suggest that sildenafil is a potential repurposable treatment for AD. However, future clinical trials are necessary to test and confirm the causal relationship of sildenafil in prevention and treatment of AD.

